# Causes of absenteeism and implementation of Regulatory Standard No. 36 in a beef processing plant: challenges in preventing musculoskeletal injuries and diseases

**DOI:** 10.47626/1679-4435-2025-1371

**Published:** 2025-07-13

**Authors:** Cintia Kaori Tomita Kikuta, Maria Luisa Alves Pereira, Driely Stephany Pedroso dos Santos, Mariana Oliveira Vicente dos Santos, Iracimara de Anchieta Messias

**Affiliations:** 1 Universidade Estadual Paulista Júlio de Mesquita Filho (UNESP), Presidente Prudente, SP, Brazil; 2 UNESP, Rio Claro, SP, Brazil

**Keywords:** ergonomics, sick leave, occupational health, occupational accidents, ergonomia, licença médica, saúde do trabalhador, acidentes de trabalho

## Abstract

**Introduction:**

Absenteeism among meat processing workers is high and mainly associated with the use of sharp hand tools, high production speeds, and adverse environmental and psychological factors.

**Objectives:**

To identify the main causes of absenteeism in a beef processing plant and evaluate the effectiveness of legal regulations, such as Regulatory Standard No. 36, in preventing absenteeism.

**Methods:**

We conducted an exploratory qualitative study to identify the causes and quantify the levels of absenteeism based on sociodemographic data and analysis of medical documents, according to the International Statistical Classification of Diseases and Related Health Problems – 10th Revision. Sick-leave data recorded by the company’s health department between 2015 and 2018 were analyzed, covering the deboning, slaughter, and evisceration operational areas. Additionally, interviews were conducted with staff members from the Safety Engineering and Occupational Medicine Department.

**Results:**

The main causes of absenteeism were injuries and external causes and musculoskeletal disorders.

**Conclusions:**

Even with the implementation of Regulatory Standard No. 36, which specifically aims to prevent accidents and illnesses in meat processing plants, the rates of absenteeism remain high. These findings indicate that legal regulations, although specific, have proven insufficient to effectively prevent health issues and ensure the well-being of workers in the Brazilian meat packing industry.

## Abstract (pt)

**Introdução:**

O absenteísmo entre trabalhadores de frigoríficos apresenta índices elevados, principalmente associados ao uso de instrumentos perfurocortantes, ao ritmo acelerado de produção e a fatores ambientais e psíquicos adversos.

**Objetivos:**

Identificar as principais causas de afastamento em um frigorífico de bovinos e avaliar a eficácia de normativas legais, como a Norma Regulamentadora nº 36, na prevenção desses afastamentos.

**Métodos:**

Trata-se de uma pesquisa exploratória, de abordagem qualitativa, que investigou os motivos e a quantidade de afastamentos com base em dados sociodemográficos e na análise de documentos médicos, conforme a Classificação Estatística Internacional de Doenças e Problemas Relacionados à Saúde – 10ª Revisão. Foram analisados dados de absenteísmo registrados pelo setor de saúde da empresa entre os anos de 2015 e 2018, abrangendo os setores de desossa, abate e miúdos. Adicionalmente, foram realizadas entrevistas com profissionais do Serviço Especializado em Engenharia de Segurança e em Medicina do Trabalho.

**Resultados:**

As principais causas de absenteísmo identificadas foram lesões e causas externas e doenças osteomusculares.

**Conclusões:**

Apesar da implementação da Norma Regulamentadora nº 36, destinada à prevenção de acidentes e doenças ocupacionais em frigoríficos, os índices de afastamento permanecem elevados. Esses achados indicam que as normativas legais, embora específicas, têm se mostrado insuficientes para assegurar a prevenção efetiva e a manutenção da saúde dos trabalhadores no setor frigorífico brasileiro.

## INTRODUCTION

Work operations in meat processing plants involve tasks requiring technical knowledge and specific skills, coupled with repetitive movements performed at high production speeds, which has alerted ergonomics professionals as it represents a public health issue.^1^ Operational roles in meat processing plants expose workers to multiple risks, compromising their comfort and safety while working.^2^ Beyond the inherent physical burden of this type of activity, there is a high demand for production, as Brazil has exported more than 1 million tons of meat and meat products annually since 2013, surpassing the export record in 2020, with 2.02 million tons.^3^

Studies^4-9^ have shown that meat processing workers are exposed to risks associated with knife use, with a high incidence of work-related musculoskeletal disorders (WMSDs) of the upper extremities. Alongside the use of sharp hand tools, the frequency of repetitive movements is high. Operations can involve up to 120 movements per minute, while ergonomics studies recommend, as a consensus, a limit of 25 to 33 movements per minute to prevent the development of musculoskeletal diseases.^10^

Regulatory Standard No. 36 (Norma Regulamentadora [NR]-36),^11^ published in 2013 with a focus on occupational health, specifically addresses health and safety in meat slaughtering and processing facilities. Among several guidelines, the most important are the limitation of repetitive movements and the implementation of job rotation, aiming to reduce illnesses and accidents.^1,4,12^ In the description of the epidemiological profile of occupational accidents occurring in a meat processing plant, a higher frequency of typical accidents has been observed, related to the characteristics of the activity performed.^13^

However, even with the implementation of NR-36, accidents and illnesses are still frequent in the meat packing industry, resulting in high rates of absenteeism.^8,14^ Therefore, this study aimed to longitudinally analyze the main causes of absenteeism and identify the operational areas with the highest absenteeism rates, even after the implementation of NR-36.

## METHODS

We conducted an exploratory qualitative study to identify the causes and quantify the levels of absenteeism over time after the implementation of NR-36. The study was carried out at a beef processing plant involved in slaughtering and processing meat for export. Our primary motivation was the persistently high absenteeism rate in operational areas even after the implementation of NR-36.

Initially, the human resources (HR) department provided us with information on the work routine, the number of active and absent employees, and the specific operational areas with the highest absenteeism rates. We then conducted interviews with staff members from the Safety Engineering and Occupational Medicine Department (Serviço Especializado em Engenharia de Segurança e em Medicina do Trabalho [SESMT]), including the occupational physician, the engineer, and the occupational safety technician. Following the interviews with HR and SESMT managers, we began analyzing documented sick-leave records.

The operational areas were cataloged, and the sick-leave documents were organized per area. Data collection covered the year 2015 and the first half of 2016. For the area with the highest incidence of absenteeism, we extended our analysis to include the entirety of 2015, 2016, 2017, and 2018 to observe the trends in the causes of absenteeism longitudinally. A total of 2,204 sick-leave records were analyzed for the year 2015 and the first half of 2016. These records allowed us to identify the number of sick-leave days paid directly by the employer (≤ 15 days of sick leave; this payment is considered part of the employee’s regular wages) and the number of cases of absenteeism (> 15 days of sick leave; where the responsibility for payment shifts to the Brazilian social security agency). Employees who were absent for reasons other than sick leave (e.g., maternity leave) during the data collection period were excluded from the analysis. Data were collected on the distribution of workers in the slaughter, deboning, and evisceration areas for 2016 according to their tenure at the company and age, and for 2020 for the slaughter and deboning sectors.

The International Statistical Classification of Diseases and Related Health Problems – 10th Revision (ICD-10) was used to categorize the reasons for sick leave, and the data were tabulated and grouped as proposed by Castro & Carvalho.^15^ This study was approved by the Research Ethics Committee of Faculdade de Ciências e Tecnologia da Universidade Estadual Paulista Júlio de Mesquita Filho (FCT/UNESP) (protocol number 3,041,653), in accordance with the guidelines outlined in Resolution No. 466/2012 of the Brazilian National Health Council.

## RESULTS

In 2015, the company had 400 employees working in its operational areas. During that year, 389 medical sick-leave certificates were issued across all operational areas. The main causes of absenteeism were injuries and external causes, for a total of 76 absences. External causes were primarily occupational accidents resulting from handling knives. Certain operational areas reported no absenteeism, including warehouse, inspection, entrance, and traceability. According to the SESMT, employees in these areas do not use hand tools, such as knives, and their tasks do not require fast pace or specific skills, which are more commonly associated with sick leaves.

The slaughter, deboning, and evisceration operational areas showed the highest absenteeism rates. This finding suggests that the type of activity performed (characterized by physically demanding operations and highly repetitive tasks with the upper extremities using tools that require specific skills, such as knives) and high production demands (i.e., organizational and psychosocial risks) can contribute to illness and accidents. NR-36 provides protocols that emphasize the importance of addressing these factors for prevention and intervention in the meat packing industry.

Given this important information, our analysis focused on data from these 3 operational areas. The slaughter, deboning, and evisceration areas employed a total of 278 workers, accounting for 70% of the company’s total operational workforce. Table 1 shows demographic data regarding sex and age in these areas.

**Table 1:** Demographic data regarding the sex and age of workers in the slaughter, deboning, and evisceration operational areas of a beef processing plant, 2015

Employees	Overall*	Slaughter	Deboning	Evisceration
Total	278	81	116	59
Men	170	74	47	33
Women	108	7	69	26
Age group (years)				
18 to 28	62	13	28	18
29 to 38	62	18	32	10
39 to 48	71	22	22	20
49 to 58	57	18	26	7
59 to 68	24	8	8	4
69 to 78	2	2	0	0
Minimum and maximum age (years)	18 to 70	20 to 70	18 to 67	19 to 63

* Total number considering workers from all 3 sectors, as reported by the human resources department.

In the slaughter area, there is a predominance of men (91.4%), while the deboning area employs a larger number of women. The predominant age group in the slaughter area is 39 to 58 years; in the deboning and evisceration areas, most workers are between 18 and 48 years of age. The slaughter area is where the production process begins, involving slaughter and carcass cleaning. The carcass is then moved to a cold room for a 24-hour aging period. Once aging is complete, the carcass is moved to the deboning area for breaking down, cutting into smaller pieces, vacuum sealing, and preparation for transport. The evisceration area handles the removal of viscera from carcasses intended for conditional use, which refers to the official destination given to raw materials and by-products, either for consumption or disposal, as outlined in Ordinance No. 392/2021.

**Figure 1.**
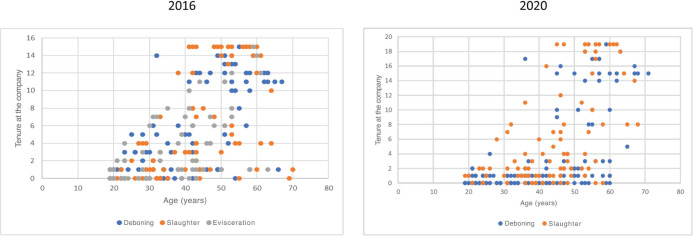
Total workforce across the slaughter, deboning, and evisceration areas in 2016 and the slaughter and deboning areas in 2020, categorized by tenure at the company (in years) and age.

It is important to identify the distribution of experienced and newly hired personnel. Figure 1 illustrates the distribution of the total workforce across the slaughter, deboning, and evisceration areas in 2016, categorized by their tenure at the company and age. For 2020, these data are available only for the slaughter and deboning areas. The slaughter area has a higher proportion of long-tenured and older workers, indicating that this area concentrates more experienced workers, which is consistent with the requirement for more skilled workers with specific knowledge to perform these tasks. In contrast, the deboning area, in 2020, showed a predominance of newly hired and younger employees. In the evisceration area in 2016, no significant differences were observed, with the presence of both newer and more experienced workers.

**Figure 2.**
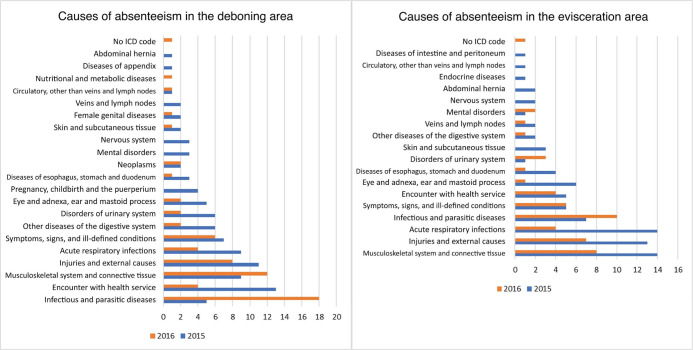
Causes of absenteeism according to the International Statistical Classification of Diseases and Related Health Problems – 10th Revision (ICD-10) in the deboning and evisceration areas, 2015 and first half of 2016.

Figure 2 illustrates the causes of absenteeism in the deboning and evisceration areas, according to ICD-10, for the year 2015 and the first half of 2016. In the first half of 2016, 89 absences were documented across the entire company. The deboning area recorded 161 absences in both periods, representing the highest absenteeism rate within the company. It is worth noting that, in both periods, infectious and parasitic diseases accounted for 40 absences, which can be explained by the considerable number of dengue fever (ICD-10 A90) cases in the region.^16^

**Figure 3.**
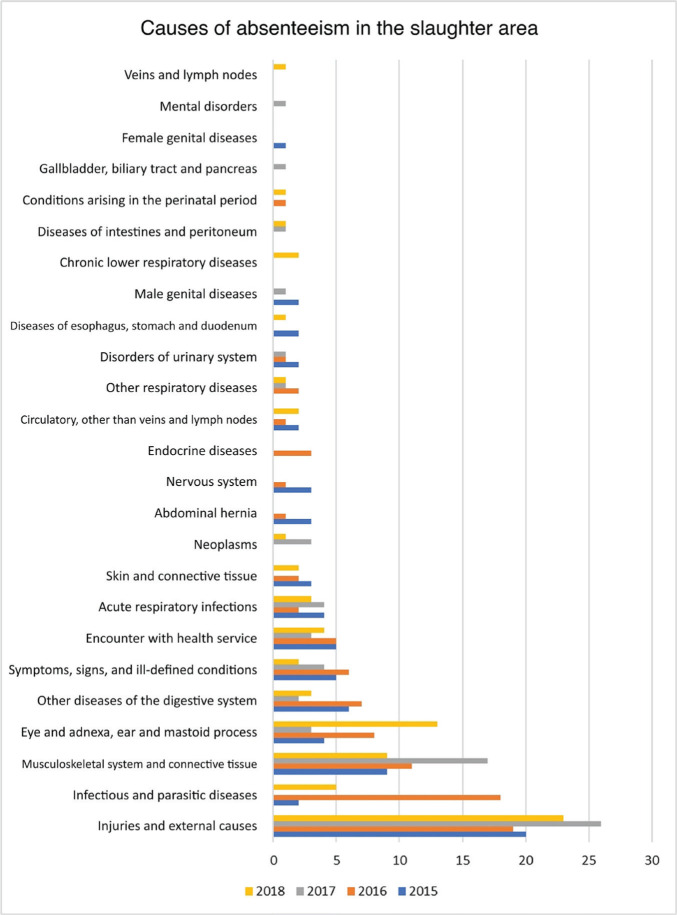
Causes of absenteeism according to the International Statistical Classification of Diseases and Related Health Problems – 10th Revision (ICD-10) in the slaughter area, 2015 to 2018.

Figure 3 shows the causes of absenteeism in the slaughter area from 2015 to 2018, as this was the area with the highest incidence of absenteeism.

In 2015, the records indicated a total of 1,176 paid sick-leave days, referring to instances where the employer directly compensated for sick leave of 15 days or less. The slaughter area reported the highest number of paid sick-leave days, with 324 days. However, as shown in Table 2, this area had the lowest number of cases of absenteeism (exceeding 15 days of sick leave) compared to the deboning and evisceration areas. In the first half of 2016, the slaughter area again reported the highest number of paid sick-leave days, totaling 191 days and surpassing the other two areas.

**Table 2:** Number of sick-leave days on certificates*, number of paid sick-leave days^†^, and number of cases of absenteeism^‡^ in the slaughter, deboning, and evisceration areas, 2015 and first half of 2016

	Slaughter	Deboning	Evisceration
2015			
Certificate days	378	267	259
Paid sick-leave days	324	173	185
Absenteeism cases	72	95	85
2016 (January to June)			
Certificate days	286	199	102
Paid sick-leave days	191	136	66
Absenteeism cases	55	94	48

* Number of sick-leave days prescribed on medical certificates received by the employer.

† Number of sick-leave days paid directly by the employer (up to 15 days of sick leave).

‡ More than 15 days of sick leave.

Upon reviewing the paid sick-leave days in the slaughter area for the full years of 2016 and 2017 (Table 3), we observed a high number of paid sick-leave days: 278 and 198, respectively. There was an increase in cases of absenteeism in 2016 compared to the previous year, which may be attributable to the dengue outbreak.

**Table 3:** Number of sick-leave days on certificates*, number of paid sick-leave days^†^, and number of cases of absenteeism^‡^ in the slaughter area, 2016 and 2017

Slaughter	n
2016 (full year)	
Certificate days	388
Paid sick-leave days	278
Absenteeism cases	89
2017 (full year)	
Certificate days	335
Paid sick-leave days	198
Absenteeism cases	69

* Number of sick-leave days prescribed on medical certificates received by the employer.

† Number of sick-leave days paid directly by the employer (up to 15 days of sick leave).

‡ More than 15 days of sick leave.

## DISCUSSION

Our study revealed that the slaughter, deboning, and evisceration areas had the highest rates of absenteeism. According to the ICD-10, the main cause of absences was injuries and external causes. In addition, these areas also showed a high number of paid sick-leave days (≤ 15 days of sick leave paid directly by the employer), totaling 1,176 days in 2015. These findings suggest that the implementation of NR-36 has not yet effectively reduced the incidence of illnesses and accidents, indicating a need for additional intervention and prevention actions, even with the current regulations.

The areas with the highest absence rates are characterized by routine knife handling during work tasks. This aligns with studies^17^ showing that the regular use of knives in meat cutting is associated with the development of WMSDs. Studies^6,18^ have also shown that the quality of knife sharpening can also influence the development of upper-extremity musculoskeletal disorders. Recommendations for limiting knife work to a maximum of 6 hours per day, combined with job rotation, have been highlighted as effective strategies.^5^ NR-36^11^ establishes that the selection of knives should be appropriate for the task and agreed upon with the worker, alongside regular knife sharpening and replacement when maintenance is insufficient to obtain adequate cutting.

NR-36^11^ also highlights job rotation as an important criterion for the prevention of WMSDs. However, for job rotation to be truly effective in preventing musculoskeletal disorders, it is essential to look beyond physical constraints and consider organizational, human, strategic, and even pedagogical factors during implementation.^1^ In this respect, implementing job rotation should be viewed as a major organizational undertaking that considers the degree of autonomy workers have to reorganize their assigned tasks (known as “margin of maneuver”).^19,20^ This margin of maneuver is essential for interventions such as job rotation to be successful in preventing WMSDs, as the opposite may expose workers to a greater risk of illness and accidents.^8,19-23^ Failure to consider these implications in regulations empirically demonstrates that simply introducing job rotation without clear guidance on how to do so is insufficient to ensure its effectiveness.^24^

The activities within the slaughter area require specific skills and physical strength, which contributes to the predominance of older, long-tenured men in this area. Their experience is valuable in maintaining production levels to meet high demand. However, physical strength is not the only determinant of quality work. Technical knowledge, specific skills, and movement variability strategies and actions are also necessary.^9^

Despite this, the slaughter area had the highest rates of absenteeism, even in the longitudinal analysis, with injuries and external causes being the main cause. This suggests that issues not covered by NR-36, such as physical characteristics, age, and experience in the activities, should be considered in the prevention of absences. Creating work debate spaces where workers can share their experiences could serve as a safety measure during work activities,^25,26^ but the involvement of different management levels is necessary to strengthen the exchange of collective knowledge, especially between older (more experienced) and younger (newer) workers.^27^

Studies indicate a trend of increasing female hires within the meat packing industry over the years.^28^ In this study, we observed that, in the deboning and evisceration areas – characterized by activities involving fine and precise manual movements –, the number of female workers is either greater than or equal to that of male workers. In this respect, absences in the deboning area due to encounters with health service may be related to gender, as women tend to seek medical care more frequently than men.^29^

In the evisceration area, the main causes of absenteeism were musculoskeletal and connective tissue diseases, injuries and external causes, and acute respiratory diseases. Public health issues also play a role in illness and absence, as evidenced by the increase in absences due to the dengue outbreak in the first half of 2016.^16^ Some studies suggest that biological waste in meat processing plants, despite not being considered an occupational hazard, can interfere with workers’ biosafety.^19^ However, our study found that musculoskeletal diseases and injuries and external causes were the prevailing causes of absence across all periods analyzed.

A larger workforce is associated with a higher rate of absenteeism, which can lead to increased costs for the company.^10^ Employees who are unable to work due to illness or injury are entitled to up to 15 days of sick leave paid directly by their employer; beyond 15 days, the responsibility for payment shifts to the Brazilian Social Security Administration (Instituto Nacional do Seguro Social, INSS), provided that the worker has a formal employment relationship under the Consolidation of Labor Laws.

In 2015 and in the first half of 2016, the slaughter area reported a higher number of paid sick-leave days (paid directly by the employer) than the deboning area, despite having fewer workers. This can be explained by the difference in the tasks performed in each area. In the slaughter area, workers not only perform repetitive cutting motions while working in a standing position, but they are also responsible for carcass cleaning, which demands a greater range of motion and physical strength.

This intense physical workload, combined with the psychological and environmental factors to which workers are exposed, contributes to illness and accidents, leading to higher rates of absenteeism. This trend is a growing concern for both public health managers and employers due to the increasing negative economic impacts.^21^ To effectively manage these impacts, it is essential to correctly diagnose the causal factors, which may not always be related to the individual worker, but rather to the working conditions and environment.^22^

While NR-36 has been implemented to protect meat packing industry workers through guidelines such as job rotation, breaks, and safe hand tool handling, absence rates due to injuries and musculoskeletal diseases remain persistently high. This suggests that the application of NR-36 alone is not sufficient to prevent or reduce occupational accidents. For prevention efforts to be truly successful, collaborative discussions and planning involving both workers and managers are crucial.^23^

Further detailed and in-depth analyses are needed to identify the sociodemographic characteristics most associated with absenteeism. This study contributes to the characterization of workers in the operational areas of a beef processing plant by identifying the areas with the highest risk of absenteeism and analyzing the underlying causes after the implementation of a specific regulation for the sector.

## CONCLUSIONS

The results of this study indicate that the rate of absenteeism in the meat packing industry due to injuries and external causes and musculoskeletal diseases is high and requires close attention. Even with the implementation of NR-36, which specifically aims to prevent accidents and illnesses in meat processing plants, WMSDs and occupational accidents remain the primary reasons for absence, especially in key operational areas: slaughter, deboning, and evisceration.

Furthermore, our data show a high incidence of short-term sick leaves (15 days or less). This not only impacts the well-being of workers but also incurs financial costs for companies through payments for days not worked.

We also observed that operational areas requiring specific knowledge and skills, such as the slaughter area, predominantly consist of older male workers with more experience in their roles. These findings suggest that professional experience may contribute to the development of preventive strategies during work tasks, and tacit knowledge gained through experience could serve as a protective factor against occupational hazards. However, further qualitative research is needed to better understand the work dynamics in this industrial sector.

Finally, while regulations are a necessary step for this industry, our data suggest that they are not sufficient to prevent health issues and ensure the well-being of workers in Brazilian meat processing plants.
